# Biapenem–fosfomycin shows enhanced *in vitro* activity compared with meropenem–fosfomycin against NDM-producing Enterobacterales

**DOI:** 10.1128/spectrum.01350-26

**Published:** 2026-10-08

**Authors:** Takashi Okanda, Daishu Fukushima, Natsuki Yumura, Niinyo Nakajima, Takuro Koshikawa, Tadatomo Oyanagi, Tomonori Takano, Tetsuo Yamaguchi, Hiroyuki Kunishima, Mitsuo Kaku, Hiromu Takemura

**Affiliations:** 1 Department of Microbiology, St. Marianna University School of Medicine 12927 , Kanagawa, Japan; 2 Department of Clinical Laboratory Technology, St. Marianna University Hospital 590207 , Kanagawa, Japan; 3 Department of Infectious Diseases, St. Marianna University School of Medicine 12927 , Kanagawa, Japan; The University of Texas at Tyler, Tyler, Texas, USA

**Keywords:** NDM-producing Enterobacterales, fosfomycin, biapenem, meropenem, checkerboard assay, time–kill assay, resistant subpopulations

## Abstract

Treatment options for New Delhi metallo-β-lactamase-producing Enterobacterales (NDME) remain limited, and the clinical relevance of fosfomycin–carbapenem combinations has not been fully established. We evaluated whether biapenem–fosfomycin provides greater *in vitro* activity than meropenem–fosfomycin. A total of 54 clinical NDME isolates (*Escherichia coli*, *n* = 22; *Klebsiella pneumoniae*, *n* = 21; *Enterobacter cloacae* complex, *n* = 11) collected in Bangladesh and Japan were analyzed. Antimicrobial susceptibility testing, checkerboard assays, checkerboard-derived cumulative inhibition analyses, time–kill assays, and assessment of fosfomycin-resistant subpopulations were performed. Biapenem exhibited lower MICs than meropenem across all species. Biapenem–fosfomycin demonstrated higher rates of synergy than meropenem–fosfomycin, particularly in *E. coli* (73% vs 45%). At concentration pairs corresponding to median minimum Fractional Inhibitory Concentration Index (mFICI) values, a greater proportion of isolates was inhibited at low biapenem concentrations in the presence of fosfomycin, whereas this effect was more limited with meropenem. At a fixed carbapenem concentration of 1 μg/mL, ≥90% inhibition was achieved at lower fosfomycin concentrations with biapenem–fosfomycin than with meropenem–fosfomycin. Time–kill analyses demonstrated that mFICI-based conditions resulted in regrowth, whereas MIC/MIC combinations of biapenem–fosfomycin maintained bacterial suppression over 24 h. Fosfomycin alone was associated with frequent recovery of fosfomycin-resistant subpopulations, whereas biapenem–fosfomycin reduced their recovery under the tested conditions. Biapenem–fosfomycin demonstrated favorable *in vitro* activity compared with meropenem–fosfomycin against NDME. These findings indicate that checkerboard-defined synergy should be interpreted together with cumulative inhibition profiles, killing kinetics, and resistant-subpopulation data.

**IMPORTANCE:**

Treating infections caused by New Delhi metallo-β-lactamase (NDM)-producing Enterobacterales remains a major global challenge because therapeutic options are limited. Fosfomycin–carbapenem combinations are frequently assessed using checkerboard-based Fractional Inhibitory Concentration Index (FICI), but this screening approach does not fully describe time-dependent killing, regrowth, or the recovery of resistant subpopulations. In this study, we compared biapenem–fosfomycin with meropenem–fosfomycin using checkerboard assays and checkerboard-derived cumulative inhibition analysis and further characterized biapenem–fosfomycin using time–kill assays and fosfomycin-resistant subpopulation assessment. Biapenem–fosfomycin showed more favorable *in vitro* activity than meropenem–fosfomycin under the tested conditions. These findings support further evaluation of biapenem–fosfomycin using dynamic pharmacokinetic models that better reproduce human drug exposure profiles.

## INTRODUCTION

New Delhi metallo-β-lactamase (NDM)-producing Enterobacterales (NDME) represent a major global therapeutic challenge because of their broad resistance profiles and limited treatment options (1). Current beta-lactam therapeutic options for NDME are largely limited to aztreonam–avibactam and cefiderocol (2). However, emerging evidence suggests that NDM enzymes may contribute to reduced susceptibility to cefiderocol, highlighting the need to further evaluate combination strategies using existing antimicrobial agents (3, 4).

Fosfomycin has re-emerged as a potential agent against multidrug-resistant gram-negative bacteria due to its unique mechanism of action and retained activity against resistant pathogens, including NDM-producing isolates (5, 6). The clinical utility of fosfomycin is limited by the rapid emergence of resistance, which is commonly associated with impaired uptake through the GlpT and UhpT transport systems, alterations in the drug target MurA, or fosfomycin-modifying enzymes, such as FosA (7–10). Combination therapy has, therefore, been proposed to enhance antibacterial activity and reduce the selection of resistant subpopulations (6).

Carbapenems have been evaluated as partner agents in combination with fosfomycin. Several studies have reported synergistic effects, particularly for meropenem-based regimens against NDM-producing *Klebsiella pneumoniae* (11–13). However, the clinical relevance of fosfomycin–meropenem combination therapy remains uncertain, as synergy assessed by fractional inhibitory concentration index (FICI) does not necessarily translate into bactericidal activity or suppression of resistance (14).

Biapenem is a carbapenem with pharmacological and structural characteristics distinct from those of meropenem, although it is less widely used internationally (15). Pharmacokinetic/pharmacodynamic (PK/PD) studies and clinical experience from Asia have supported its use in selected infectious disease settings (15–17). In our previous study, a biapenem-based combination demonstrated enhanced *in vitro* activity against cefiderocol-resistant NDME, supporting further evaluation of biapenem as a partner agent in combination strategies (18). However, biapenem–fosfomycin combinations have not been systematically compared with meropenem–fosfomycin against NDME.

In this study, we evaluated the *in vitro* activity of biapenem–fosfomycin in comparison with meropenem–fosfomycin against NDME using clinical isolates collected from Bangladesh and Japan.

## MATERIALS AND METHODS

### Bacterial isolates

A total of 54 non-duplicate NDME isolates were included in this study, comprising *Escherichia coli* (*n* = 22), *K. pneumoniae* (*n* = 21), and *Enterobacter cloacae* complex (*n* = 11). These isolates harbored *bla*_NDM-1_ (*n* = 24), *bla*_NDM-5_ (*n* = 19), or *bla*_NDM-7_ (*n* = 11). A total of 41 isolates were collected in Bangladesh between 2015 and 2017, and 13 isolates were collected in Japan between 2018 and 2020. Isolates were recovered from blood, respiratory tract specimens, urine, and other clinical specimens, including normally sterile sites. All isolates were stored at −80°C in tryptic soy broth supplemented with 20% glycerol until analysis. Species identification was performed using the MALDI Biotyper System (Bruker Daltonics, Billerica, MA, USA). Carbapenemase production was confirmed by the modified carbapenem inactivation method, and β-lactamase-encoding genes were determined by PCR and Sanger sequencing as previously described (19). Multilocus sequence typing (MLST) was performed for all isolates using species-specific schemes: PubMLST for *E. coli* and *E. cloacae* complex and the Institut Pasteur BIGSdb database for *K. pneumoniae*. Isolate-level sequence types, NDM variants, country and year of isolation, specimen sources, and co-carried β-lactamase gene profiles are summarized in Table S1.

### Antimicrobial susceptibility testing

MICs of biapenem (LKT Laboratories Inc., USA) and meropenem (LKT Laboratories Inc.) were determined by broth microdilution (BMD) according to Clinical and Laboratory Standards Institute (CLSI) guidelines (20). Fosfomycin (FUJIFILM Wako Chemicals, Japan) MICs were determined by both BMD and agar dilution (AD) according to CLSI M07, with glucose-6-phosphate (G6P, Sigma-Aldrich, Co., USA) supplementation at 25 μg/mL in both assays (20). AD was considered the reference method for fosfomycin susceptibility. All MIC measurements were performed in triplicate, and median values were used for analysis. *E. coli* ATCC 25922 was used for quality control.

### Checkerboard assay

Checkerboard assays were performed on 54 isolates using twofold serial dilutions of fosfomycin (0.03–1,024 μg/mL) and carbapenems (biapenem: 0.03–512 μg/mL, meropenem: 0.03–2048 μg/mL) using cation-adjusted Mueller–Hinton broth (CAMHB, Becton, Dickinson and Co., USA) supplemented with G6P at 25 μg/mL. The final bacterial inoculum was approximately 5 × 10^5^ CFU/mL. Plates were incubated at 35 ± 2°C for 24 h, and growth was assessed by visual inspection. *E. coli* ATCC 25922 was used for quality control. The fractional inhibitory concentration index (FICI) was calculated as FICI = (MICA in combination/MICA alone) + (MICB in combination/MICB alone). FICI was calculated for all concentration combinations showing no visible growth. For each isolate, the minimum FICI (mFICI) was defined as the lowest FICI among all no-growth wells.

To evaluate population-level inhibitory activity, checkerboard-derived cumulative inhibition heatmaps were generated. The proportion of inhibited isolates was calculated as the number of isolates showing no visible growth divided by the total number of isolates tested within each species and expressed as a percentage.

### Time–kill assays

Time–kill assays were performed using nine representative isolates consisting of one NDM-1-, one NDM-5-, and one NDM-7-producing isolate from each species. For each species–NDM variant combination, isolates were selected based on low mFICI values for the biapenem–fosfomycin combination and baseline MICs of biapenem and fosfomycin that were not excessively high, allowing evaluation under interpretable concentration conditions.

Time–kill assays were performed using five experimental conditions: no drug, fosfomycin alone at MIC, biapenem alone at MIC, biapenem–fosfomycin at the mFICI pair, and biapenem–fosfomycin at the MIC/MIC pair. The mFICI pair was defined as the concentration combination at which the minimum FICI was observed in the checkerboard assay. The MIC/MIC pair was defined as biapenem and fosfomycin each tested at their respective MICs determined by BMD. Bacterial suspensions were prepared in CAMHB supplemented with G6P at 25 μg/mL to a final inoculum of approximately 1 × 10^6^ CFU/mL. Each assay was performed in a final volume of 10 mL and incubated with shaking at 35 ± 2°C. At 0, 3, 6, and 24 h after antimicrobial exposure, 100 μL aliquots were collected and serially diluted, and 100 μL of each dilution was plated onto tryptic soy agar. Plates were incubated at 35 ± 2°C for 24 h, and viable bacterial counts were calculated from colony counts. To minimize antibiotic carryover effects, samples were serially diluted before plating. The lower limit of detection was 10 CFU/mL.

To assess the impact of bacterial burden, time–kill assays were additionally performed for *K. pneumoniae* isolates using a 10-fold higher initial inoculum. Experimental conditions and sampling time points were identical to those described above. *K. pneumoniae* was selected for this exploratory analysis because it is a clinically important NDM-producing pathogen and has been the focus of previous fosfomycin–carbapenem combination studies.

### Determination of fosfomycin-resistant subpopulations

To evaluate the emergence of fosfomycin-resistant subpopulations, cultures obtained after 24 h of time–kill assays were plated onto drug-free agar and agar containing fosfomycin at eight times the baseline MIC determined by AD, with G6P supplementation at 25 μg/mL. Colonies on fosfomycin-containing agar were counted after 48 h of incubation at 35 ± 2°C. The frequency of resistant subpopulations was calculated as the ratio of colony counts recovered on fosfomycin-containing agar (R) to those on drug-free agar (N) expressed as R/N. A value of 0 indicates no detectable colonies under the given conditions.

### Data analysis

BMD-derived MICs were used for checkerboard and time–kill assays to ensure consistency across liquid-phase *in vitro* assays, whereas AD was used as the reference method for fosfomycin susceptibility interpretation. Synergistic interactions were defined as FICI ≤ 0.5, no interaction as >0.5–4.0, and antagonism as >4.0 (21). Synergy in time–kill assays was defined as a ≥2 log_10_ CFU/mL reduction with the combination compared with the most active single agent at 24 h, and bactericidal activity in time–kill assays was defined as a ≥3 log_10_ CFU/mL reduction from the initial inoculum (22). Categorical susceptibility interpretations were applied only to single-agent MICs for which CLSI criteria were available (23).

## RESULTS

### Antimicrobial susceptibility profiles

A total of 54 NDME isolates, including *E. coli* (*n* = 22), *K. pneumoniae* (*n* = 21), and *E. cloacae* complex (*n* = 11), were analyzed. Biapenem exhibited consistently lower MIC values than meropenem across all species (Table 1, Fig. 1). The MIC_50_/MIC_90_ values of biapenem were 4/16 μg/mL for *E. coli*, 4/16 μg/mL for *K. pneumoniae*, and 1/4 μg/mL for *E. cloacae* complex, whereas those of meropenem were 128/128, 64/128, and 16/128 μg/mL, respectively. Fosfomycin MICs determined by AD were higher than those obtained by BMD, particularly in *K. pneumoniae* and *E. cloacae* complex (Table 1).

**TABLE 1:** *In vitro* antimicrobial susceptibility of NDM-producing Enterobacterales isolates^*a*^

Species (*n*)	Antimicrobial agent	MIC range (μg/mL)	MIC_50_	MIC_90_
*E. coli* (22)	BPM	1–16	4	16
	MEM	16–128	128	128
	FOF (BMD)	2–16	4	16
	FOF (AD)	4–256	32	64
*K. pneumoniae* (21)	BPM	1–256	4	16
	MEM	8–1,024	64	128
	FOF (BMD)	64–512	256	512
	FOF (AD)	512–4,096	1,024	4,096
*E. cloacae* complex (11)	BPM	0.25–8	1	4
	MEM	2–128	16	128
	FOF (BMD)	4–256	64	128
	FOF (AD)	32–2,048	512	1,024

^*a*^ Fosfomycin MICs were determined by broth microdilution (BMD) and agar dilution (AD) with glucose-6-phosphate; agar dilution was considered the reference method. BPM, biapenem; MEM, meropenem; FOF, fosfomycin; BMD, broth microdilution; AD, agar dilution; MIC, minimum inhibitory concentration.

**Fig 1:**
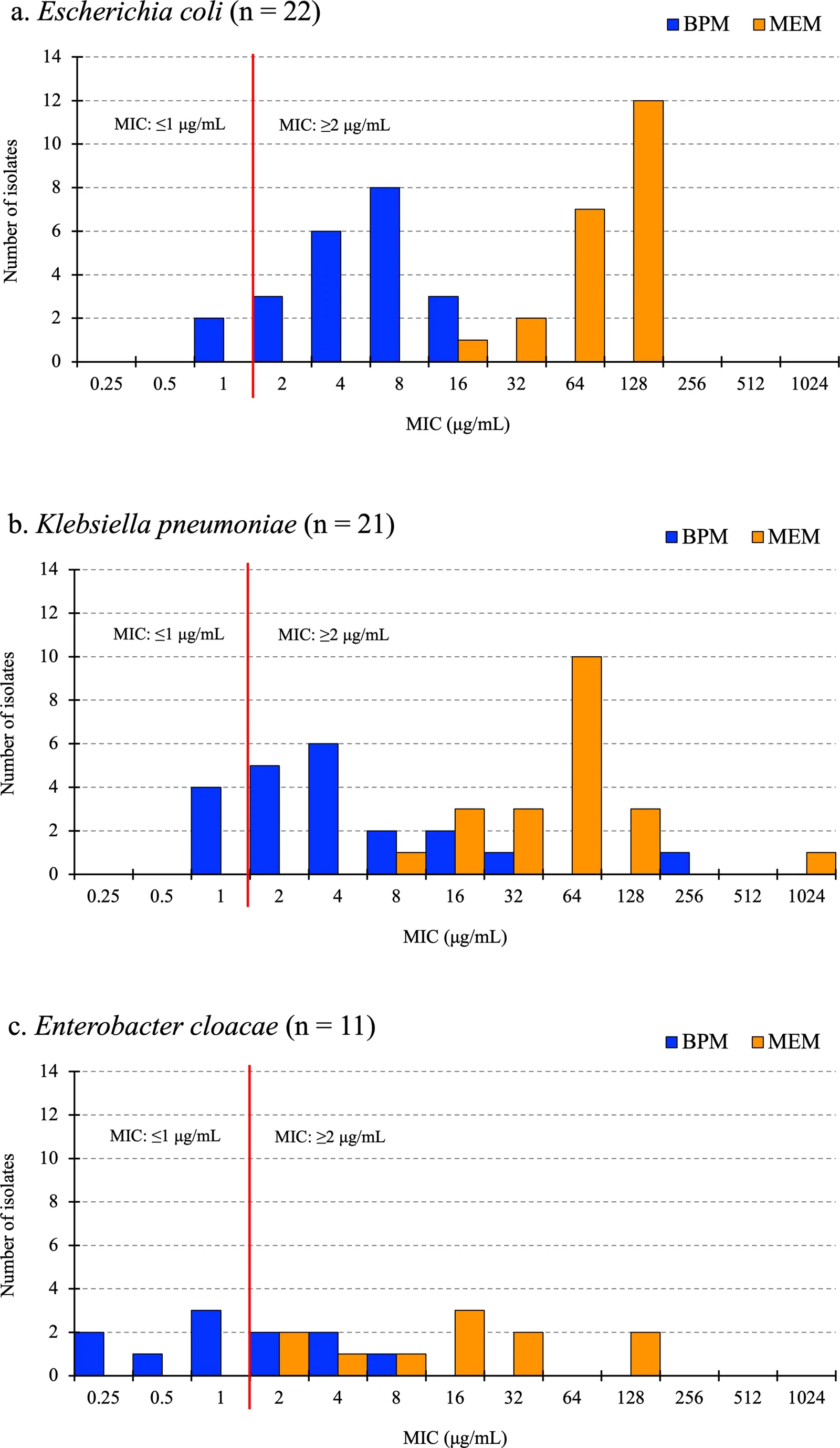
MIC distributions of biapenem and meropenem in NDM-producing Enterobacterales isolates. Panels show MIC distributions for (a) *Escherichia coli*, (b) *Klebsiella pneumoniae*, and (c) *Enterobacter cloacae* complex. Bars represent the number of isolates at each MIC value. The vertical red line indicates 1 μg/mL corresponding to the CLSI susceptibility breakpoint for meropenem (MEM). For biapenem (BPM), this concentration was used only as a reference concentration for comparative *in vitro* analysis and not to assign clinical susceptibility categories.

### Synergistic activity of fosfomycin–carbapenem combinations

The distribution of mFICI values is shown in Fig. 2. For biapenem–fosfomycin, the proportion of isolates with mFICI ≤0.5 was 16/22 (73%) in *E. coli*, 21/21 (100%) in *K. pneumoniae*, and 11/11 (100%) in *E. cloacae* complex, with median mFICI values of 0.375, 0.250, and 0.313, respectively. For meropenem–fosfomycin, corresponding proportions were 10/22 (45%), 20/21 (95%), and 10/11 (91%), with median mFICI values of 0.516, 0.250, and 0.250, respectively.

**Fig 2:**
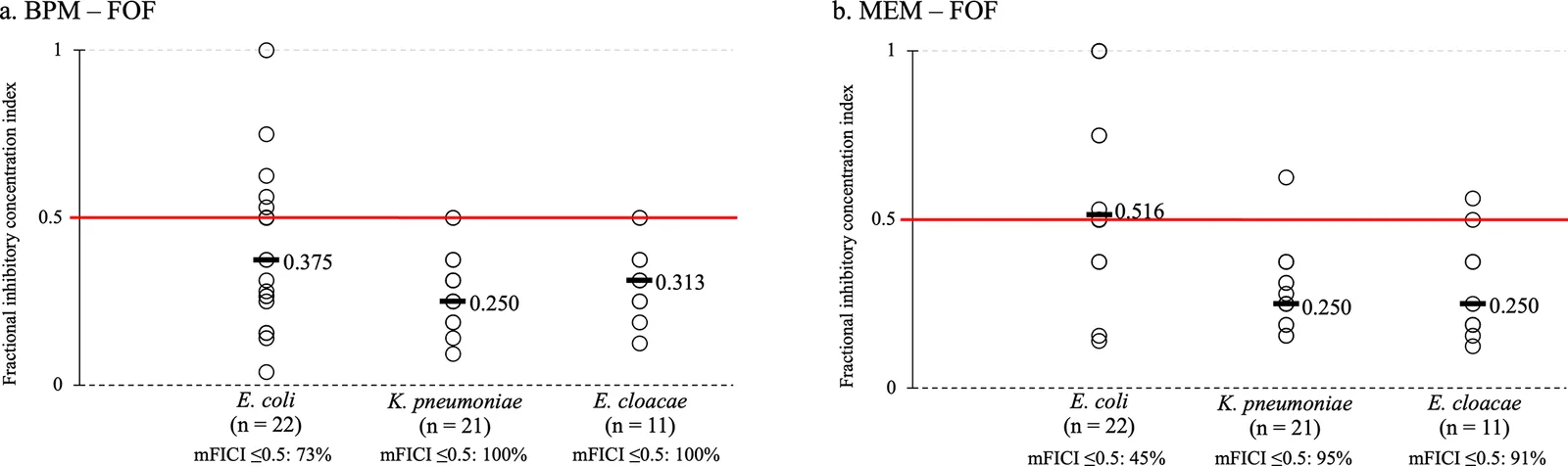
The minimum fractional inhibitory concentration index (mFICI) distributions for biapenem–fosfomycin and meropenem–fosfomycin combinations. Panels show mFICI distributions for (a) biapenem–fosfomycin and (b) meropenem–fosfomycin. Each point represents mFICI for an individual isolate. Horizontal lines indicate median values. The red line represents the threshold for synergy (mFICI ≤ 0.5). Synergy was defined as an mFICI ≤ 0.5. BPM, biapenem; MEM, meropenem; FOF, fosfomycin.

### Inhibition at low carbapenem concentrations in combination with fosfomycin

The proportion of isolates inhibited at carbapenem concentrations of ≤1 µg/mL, alone or in combination with fosfomycin at median mFICI concentrations, is shown in Fig. 3. For biapenem, the proportion of inhibited isolates increased from 9 to 73% in *E. coli*, 19 to 86% in *K. pneumoniae*, and 55 to 100% in *E. cloacae* complex. For meropenem, the corresponding proportions remained 0% in *E. coli* and increased from 0 to 19% in *K. pneumoniae* and 0 to 55% in *E. cloacae* complex.

**Fig 3:**
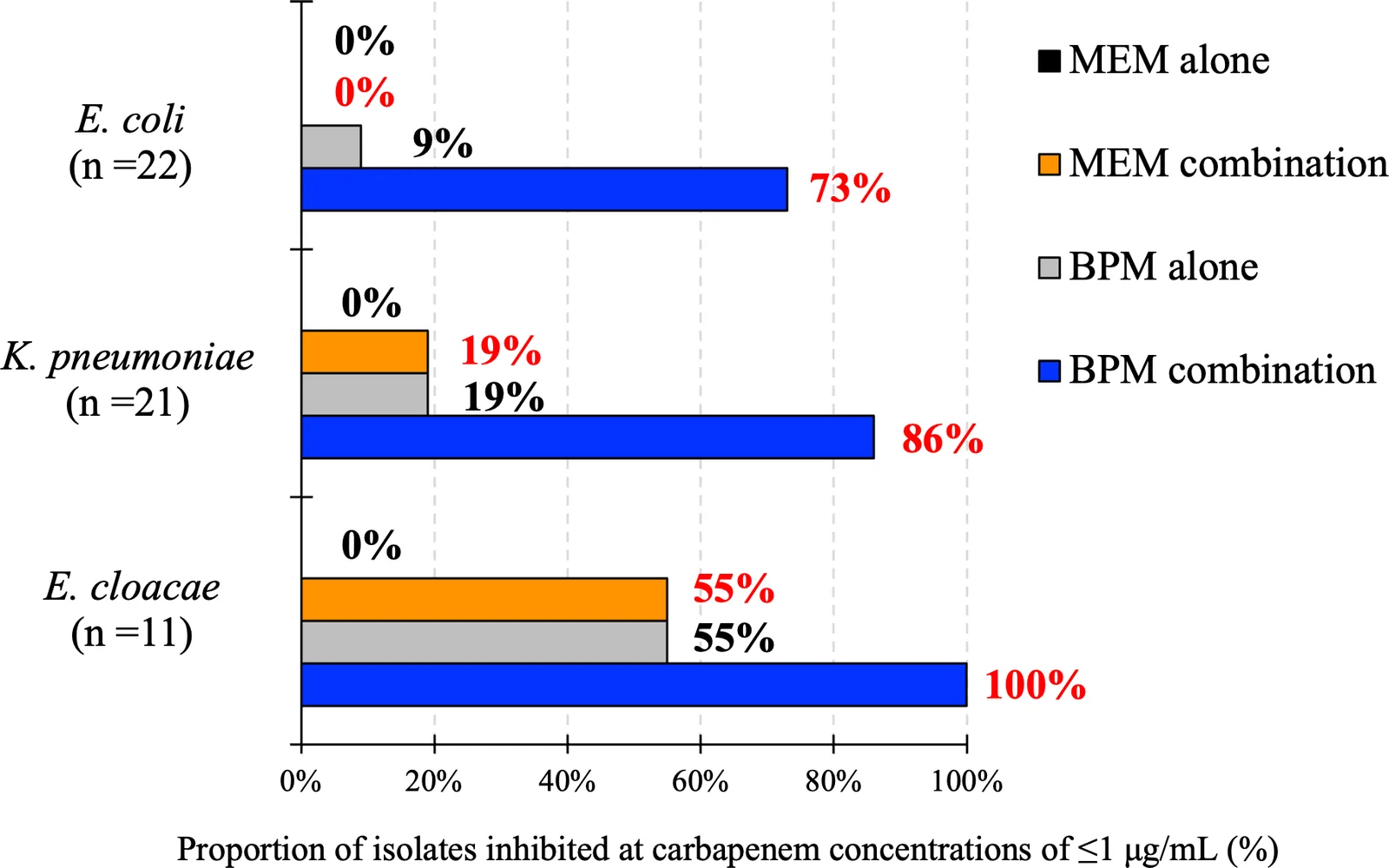
Proportion of isolates inhibited at low carbapenem concentrations alone or in combination with fosfomycin. Bars indicate the proportion of isolates showing no visible growth at carbapenem concentrations ≤ 1 µg/mL, alone or in combination with fosfomycin at median mFICI concentrations. This concentration corresponds to the CLSI susceptibility breakpoint for meropenem and is used only as a reference concentration for comparative *in vitro* analysis. It was not used to assign clinical susceptibility categories to biapenem or to combination regimens. BPM, biapenem; MEM, meropenem; FOF, fosfomycin; mFICI, minimum Fractional Inhibitory Concentration Index.

### Checkerboard-derived cumulative inhibition analysis

Cumulative inhibition heatmaps are shown in Fig. 4. At a carbapenem concentration of 1 μg/mL, ≥90% inhibition was achieved with fosfomycin concentrations of 4 (*E. coli*), 64 (*K. pneumoniae*), and 16 μg/mL (*E. cloacae* complex) for biapenem–fosfomycin. In contrast, meropenem–fosfomycin required higher fosfomycin concentrations (16, 256, and 128 μg/mL, respectively) to achieve similar inhibition.

**Fig 4:**
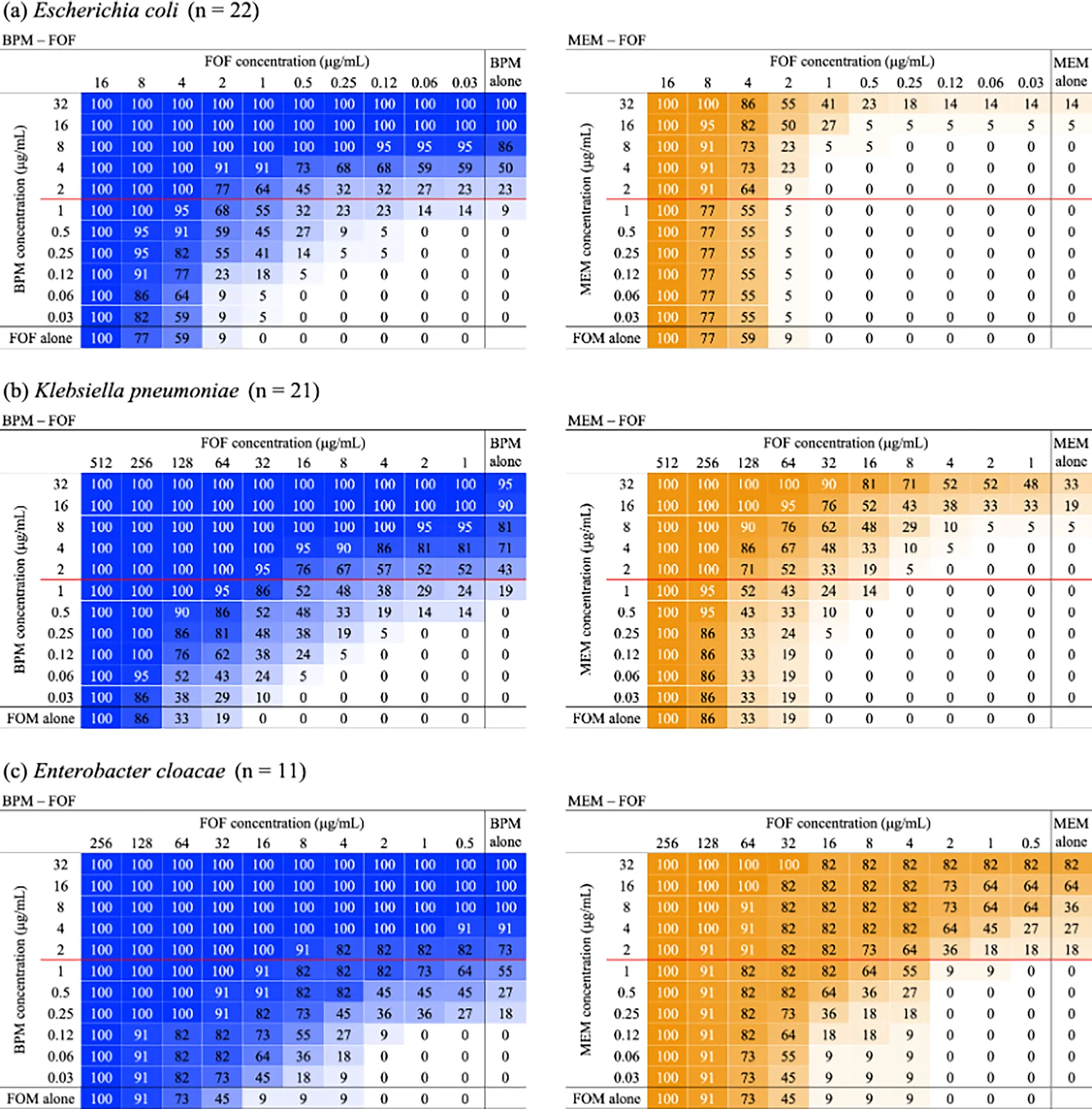
Checkerboard-derived cumulative inhibition heatmaps for biapenem–fosfomycin and meropenem–fosfomycin combinations. Panels show checkerboard-derived cumulative inhibition heatmaps for (a) *Escherichia coli*, (b) *Klebsiella pneumoniae*, and (c) *Enterobacter cloacae* complex. Heatmaps represent the cumulative percentage of isolates inhibited at each combination of carbapenem and fosfomycin concentrations. Rows indicate carbapenem concentrations, and columns indicate fosfomycin concentrations. Values correspond to the proportion (%) of isolates showing no visible growth. The horizontal red line indicates a carbapenem concentration of 1 μg/mL corresponding to the CLSI susceptibility breakpoint for meropenem. For biapenem, this concentration was used only as a reference concentration for comparative *in vitro* analysis and not to assign clinical susceptibility categories. BPM, biapenem; MEM, meropenem; FOF, fosfomycin.

### Time–kill analysis

Time–kill assays were performed using representative isolates producing NDM-1, NDM-5, or NDM-7 (Fig. 5). At mFICI concentrations, modest reductions at 6 h were followed by regrowth at 24 h, with bacterial counts reaching 1.4 × 10^9^ to 2.3 × 10^9^ CFU/mL in *E. coli*, 2.2 × 10^8^ to 2.5 × 10^9^ CFU/mL in *K. pneumoniae*, and 3.0 × 10^8^ to 3.4 × 10^9^ CFU/mL in *E. cloacae* complex. At MIC/MIC concentrations, bacterial counts were markedly reduced (≤10^3^ CFU/mL at 6 h) and remained low at 24 h, ranging from 36 to 4.0 × 10^4^ CFU/mL in *E. coli*, 36 to 260 CFU/mL in *K. pneumoniae*, and 36 to 3.6 × 10^3^ CFU/mL in *E. cloacae* complex. Under high-inoculum conditions in *K. pneumoniae*, reduced activity was observed, with viable counts of 6.2 × 10^3^ to 5.4 × 10^4^ CFU/mL at 6 h and 2.6 × 10^5^ to 5.1 × 10^6^ CFU/mL at 24 h.

**Fig 5:**
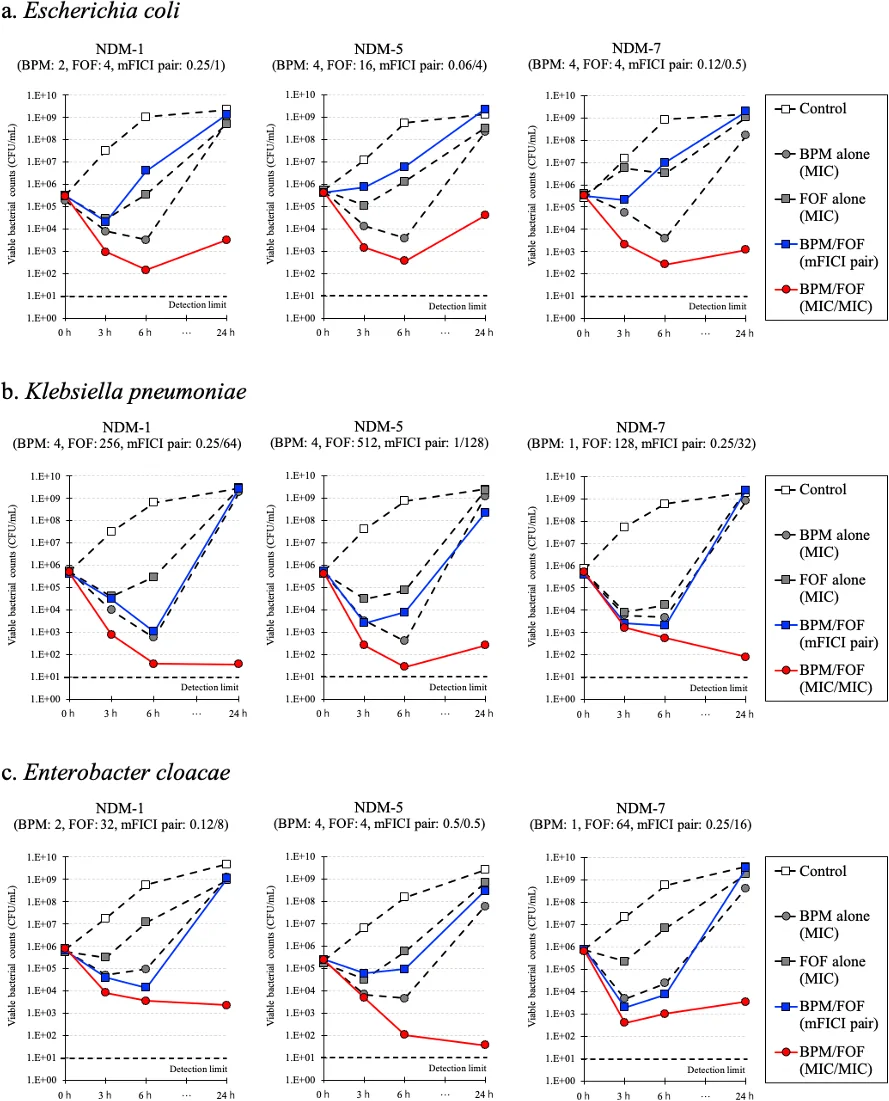
Time–kill kinetics of biapenem–fosfomycin combinations against NDM-producing Enterobacterales. Panels show time–kill kinetics for (a) *Escherichia coli*, (b) *Klebsiella pneumoniae*, and (c) *Enterobacter cloacae* complex; each panel includes representative NDM-1-, NDM-5-, and NDM-7-producing isolates. Bacterial counts (CFU/mL) were determined at 0, 3, 6, and 24 h. Biapenem and fosfomycin were tested alone at their respective MICs and in combination at concentrations corresponding to the mFICI pair and the MIC/MIC pair. Bactericidal activity was defined as a ≥3 log10 reduction in CFU/mL from the initial inoculum. The dashed horizontal line indicates the limit of detection. BPM, biapenem; FOF, fosfomycin; MIC, minimum inhibitory concentration; mFICI, minimum fractional inhibitory concentration index; CFU, colony-forming units.

### Emergence of fosfomycin-resistant subpopulations

The frequency of fosfomycin-resistant subpopulations after 24 h antimicrobial exposure, calculated from colony counts after 48 h incubation on fosfomycin-containing agar, is shown in Table 2. Fosfomycin alone yielded the highest frequencies of fosfomycin-resistant subpopulations, ranging from 1.3 × 10^−2^ to 9.6 × 10^−2^ in *E. coli*, 8.1 × 10^−5^ to 2.3 × 10^−4^ in *K. pneumoniae*, and 2.7 × 10^−4^ to 3.6 × 10^−4^ in *E. cloacae* complex. Biapenem alone did not eliminate fosfomycin-resistant subpopulations, whereas biapenem–fosfomycin was associated with reduced recovery of fosfomycin-resistant subpopulations under the tested conditions. At mFICI concentrations, frequencies decreased to 4.5 × 10^−7^ to 3.6 × 10^−5^ (*E. coli*) and 1.0 × 10^−7^ to 9.1 × 10^−6^ (*K. pneumoniae*) and were ≤10^−5^ or undetectable in *E. cloacae* complex. At MIC/MIC concentrations, resistant colonies were not detected in any of the tested isolates across all species.

**TABLE 2:** Characteristics, MICs, and frequency of fosfomycin-resistant subpopulations in representative isolates^*a*^

Isolate ID	Species	NDM variant	BPM MIC	FOF MIC (BMD)	FOF MIC (AD)	Screening FOF conc. (8× MIC)	Frequency of fosfomycin-resistant subpopulations (R/N) at 48 h			
							BPM alone (MIC)	FOF alone (MIC)	BPM/FOF (mFICI)	BPM/FOF (MIC/MIC)
SMU6508	*E. coli*	NDM-1	2	4	16	128	1.5 × 10^−4^	9.6 × 10^−2^	3.6 × 10^−5^	0
SMU6510	*E. coli*	NDM-5	4	16	32	256	9.1 × 10^−4^	1.3 × 10^−2^	4.5 × 10^−7^	0
SMU6412	*E. coli*	NDM-7	4	4	64	512	1.2 × 10^−3^	2.6 × 10^−2^	2.5 × 10^−5^	0
SMU6421	*K. pneumoniae*	NDM-1	4	256	1024	8192	2.6 × 10^−7^	1.8 × 10^−5^	2.7 × 10^−6^	0
SMU6424	*K. pneumoniae*	NDM-5	4	512	1024	8192	1.2 × 10^−5^	2.3 × 10^−4^	9.1 × 10^−6^	0
SMU6428	*K. pneumoniae*	NDM-7	1	128	512	4096	0	8.1 × 10^−5^	1.0 × 10^−7^	0
SMU6589	*E. cloacae* complex	NDM-1	2	32	256	2048	4.2 × 10^−7^	3.6 × 10^−4^	0	0
SMU6458	*E. cloacae* complex	NDM-5	4	4	32	256	0	2.9 × 10^−4^	0	0
SMU6590	*E. cloacae* complex	NDM-7	1	64	256	2048	0	2.7 × 10^−4^	1.8 × 10^−5^	0

^*a*^ Frequency of fosfomycin-resistant subpopulations (R/N) at 48 h of incubation was calculated as the ratio of colony counts recovered on fosfomycin-containing agar (R) to those on drug-free agar (N) after 24 h exposure in time–kill assays. Fosfomycin concentrations used for selection were 8× the baseline MIC determined by AD. A value of 0 indicates no detectable colonies under the given conditions. BPM, biapenem; FOF, fosfomycin; BMD, broth microdilution; AD, agar dilution; mFICI, minimum fractional inhibitory concentration index; R/N, ratio of colony counts recovered on fosfomycin-containing agar to those on drug-free agar.

## DISCUSSION

In this study, biapenem–fosfomycin showed more favorable *in vitro* activity than meropenem–fosfomycin against NDME in checkerboard-based mFICI and cumulative inhibition analyses. Time–kill assays and assessment of fosfomycin-resistant subpopulations further characterized the killing kinetics, regrowth, and resistance-related behavior of biapenem–fosfomycin under the tested static *in vitro* conditions.

The activity of fosfomycin–carbapenem combinations varied among species. Strong synergistic effects were observed in *K. pneumoniae*, consistent with previous reports of fosfomycin–meropenem activity against NDM-producing *K. pneumoniae* (11–13). However, the magnitude of interaction and the recovery of fosfomycin-resistant subpopulations differed among *E. coli*, *K. pneumoniae*, and *E. cloacae* complex. These differences may reflect species-dependent variation in fosfomycin uptake, intrinsic resistance mechanisms, and baseline carbapenem MICs. Chromosomal FosA-like enzymes in *Klebsiella* spp. may contribute to reduced fosfomycin activity, although this mechanism was not directly examined in the present study (10).

Biapenem exhibited lower baseline MICs than meropenem across the isolate collection. This difference likely contributed to the greater proportion of isolates inhibited at low biapenem concentrations in the presence of fosfomycin. We, therefore, interpret the checkerboard-derived results as *in vitro* inhibition profiles rather than changes in clinical susceptibility categories. Although a carbapenem concentration of ≤1 µg/mL was used as a reference concentration for comparative analysis, this should not be interpreted as a validated breakpoint for biapenem or for combination regimens. The mechanism underlying the lower biapenem MICs compared with meropenem was not investigated and may involve differences in enzyme–substrate interactions, permeability, or other bacterial factors.

Checkerboard-derived cumulative inhibition profiles provided additional context for interpreting FICI-based synergy, but FICI itself should be interpreted as a screening metric. The use of mFICI identifies the lowest FICI among no-growth wells and is, therefore, useful for detecting potential interactions, but it does not describe time-dependent killing or regrowth (14). The checkerboard-derived cumulative inhibition analysis helped contextualize the population-level activity of each concentration pair and showed that biapenem–fosfomycin inhibited a higher proportion of isolates at lower fosfomycin concentrations than meropenem–fosfomycin. These findings support the comparative *in vitro* advantage of biapenem–fosfomycin under the tested conditions; however, they should be interpreted as static *in vitro* inhibition profiles rather than evidence of clinical susceptibility.

Time–kill assays provided additional information regarding killing dynamics. Under mFICI-based conditions, initial reductions were followed by regrowth at 24 h, whereas MIC/MIC combinations of biapenem–fosfomycin maintained bacterial suppression over 24 h. Together, these findings indicate that checkerboard-defined interaction, cumulative inhibition, and time-dependent killing kinetics should be interpreted together. Under high-inoculum conditions in *K. pneumoniae*, reduced activity was observed. This finding should be interpreted as an *in vitro* observation, and its clinical relevance remains uncertain (24).

Fosfomycin-resistant subpopulations were recovered more frequently after fosfomycin exposure alone, whereas biapenem–fosfomycin was associated with reduced recovery of these subpopulations under the tested static *in vitro* conditions. This finding is consistent with the known limitation of fosfomycin monotherapy, namely, the selection of resistant subpopulations (25, 26). These data do not define a specific exposure target for resistance suppression. Rather, they indicate that the tested biapenem–fosfomycin combination conditions reduced recovery of fosfomycin-resistant subpopulations compared with fosfomycin alone.

This study has several limitations. First, all combination experiments were performed using static *in vitro* drug concentrations and, therefore, did not reproduce the dynamic PK profiles observed in patients. The static concentrations used in checkerboard and time–kill assays should not be interpreted as directly replicable serum exposures. Future studies using dynamic models, such as hollow-fiber infection models, are needed to evaluate whether the observed *in vitro* activity is maintained under human-like PK conditions. Second, time–kill assays and resistant-subpopulation analyses were performed using a limited number of representative isolates, although they were selected to include multiple species and NDM variants. Third, no validated clinical breakpoint exists for biapenem, and combination-specific breakpoints are not established. Therefore, combination results were interpreted as *in vitro* inhibition profiles rather than changes in clinical susceptibility categories. Finally, the mechanisms underlying the observed interaction were not examined and require further investigation.

In conclusion, biapenem–fosfomycin showed favorable *in vitro* activity compared with meropenem–fosfomycin against NDME in checkerboard and cumulative inhibition analyzes. Time–kill assays and resistant-subpopulation assessment further supported the *in vitro* activity of biapenem–fosfomycin under the tested static conditions. Further studies using dynamic PK models are warranted to evaluate whether these findings are maintained under clinically relevant dynamic exposure conditions.

## Data Availability

All data supporting the findings of this study are included in the article and its Supplemental material. The underlying numerical data sets are available from the corresponding author upon request.

## SUPPLEMENTAL MATERIAL

Table S1 spectrum.01350-26-s0001.docx Sequence types and co-carried β-lactamase gene profiles of NDME.

ASM does not own the copyrights to Supplemental Material that may be linked to, or accessed through, an article. The authors have granted ASM a non-exclusive, world-wide license to publish the Supplemental Material files. Please contact the corresponding author directly for reuse.
