# Supplementary material for: Biapenem–fosfomycin shows enhanced in vitro activity compared with meropenem–fosfomycin against NDM-producing Enterobacterales
Source: Microbiol Spectr. 2026 Oct 8;14(11):e01350-26. doi: 10.1128/spectrum.01350-26 (PMC13729167; doi:10.1128/spectrum.01350-26)
Supplement: Supplementary file 1 [file spectrum.01350-26-s0001.docx]

**Supplementary Table S1.** Sequence types and co-carried β-lactamase gene profiles of NDM-producing Enterobacterales isolates used in this study

| Species | NDM variant | Isolate ID | Sequence type | Co-carried β-lactamase genes other than *bla*_NDM_ | Country | Year | Specimen source |
| --- | --- | --- | --- | --- | --- | --- | --- |
| *E. coli* | NDM-1 | SMU6656 | ST10 | TEM-1B + SHV-12 + CTX-M-15 | Bangladesh | 2017 | Other |
| *E. coli* | NDM-1 | SMU6546 | ST69 | – | Japan | 2018 | Blood |
| *E. coli* | NDM-1 | SMU6492 | ST101 | TEM-1A + CTX-M-15 + CMY-4 | Bangladesh | 2017 | Blood |
| *E. coli* | NDM-1 | SMU6513 | ST648 | TEM-1A + TEM-1B + CTX-M-15 | Bangladesh | 2017 | Blood |
| *E. coli* | NDM-1 | SMU6508* | ST8346 | TEM-1B + CTX-M-15 + CMY-42 | Bangladesh | 2017 | Blood |
| *E. coli* | NDM-5 | SMU6545 | ST10 | TEM-1B + CTX-M-15 | Japan | 2018 | Blood |
| *E. coli* | NDM-5 | SMU6500 | ST68 | CTX-M-15 | Bangladesh | 2017 | Blood |
| *E. coli* | NDM-5 | SMU6497 | ST101 | TEM-1B + CTX-M-15 | Bangladesh | 2017 | Blood |
| *E. coli* | NDM-5 | SMU6503 | ST167 | TEM-1B + CMY-42 | Bangladesh | 2017 | Blood |
| *E. coli* | NDM-5 | SMU6547 | ST167 | CTX-M-15 | Japan | 2020 | Blood |
| *E. coli* | NDM-5 | SMU6600 | ST167 | CTX-M-15 | Japan | 2020 | Blood |
| *E. coli* | NDM-5 | SMU6502 | ST405 | TEM-1B + CMY-42 | Bangladesh | 2017 | Blood |
| *E. coli* | NDM-5 | SMU6406 | ST648 | TEM-1B + CTX-M-15 + CMY-42 | Bangladesh | 2015 | Urine |
| *E. coli* | NDM-5 | SMU6415 | ST648 | TEM-1B + CTX-M-15 + CMY-42 | Bangladesh | 2015 | Urine |
| *E. coli* | NDM-5 | SMU6548 | ST648 | TEM-1B + CTX-M-15 | Japan | 2020 | Blood |
| *E. coli* | NDM-5 | SMU6510* | ST2659 | TEM-1B | Bangladesh | 2017 | Blood |
| *E. coli* | NDM-7 | SMU6414 | ST101 | TEM-1B + CTX-M-15 | Bangladesh | 2015 | Urine |
| *E. coli* | NDM-7 | SMU6512 | ST205 | CTX-M-15 | Bangladesh | 2017 | Blood |
| *E. coli* | NDM-7 | SMU6412* | ST405 | TEM-1B + CTX-M-15 | Bangladesh | 2015 | Urine |
| *E. coli* | NDM-7 | SMU6413 | ST448 | TEM-1B | Bangladesh | 2015 | Urine |
| *E. coli* | NDM-7 | SMU6504 | ST448 | CMY-42 | Bangladesh | 2017 | Blood |
| *E. coli* | NDM-7 | SMU6544 | ST448 | TEM-1B | Japan | 2018 | Blood |
| *K. pneumoniae* | NDM-1 | SMU6514 | ST11 | TEM-1B + SHV-158 + CTX-M-15 | Bangladesh | 2017 | Respiratory tract |
| *K. pneumoniae* | NDM-1 | SMU6517 | ST11 | TEM-1A + SHV-158 + CTX-M-15 | Bangladesh | 2017 | Urine |
| *K. pneumoniae* | NDM-1 | SMU6425 | ST34 | TEM-1B + SHV-26 + CTX-M-15 + DHA-1 | Bangladesh | 2015 | Blood |
| *K. pneumoniae* | NDM-1 | SMU6426 | ST34 | TEM-1B + SHV-26 + CTX-M-15 + DHA-1 | Bangladesh | 2015 | Urine |
| *K. pneumoniae* | NDM-1 | SMU6427 | ST34 | TEM-1B + SHV-26 + CTX-M-15 + DHA-1 | Bangladesh | 2015 | Blood |
| *K. pneumoniae* | NDM-1 | SMU6432 | ST34 | TEM-1B + SHV-26 + CTX-M-15 + DHA-1 | Bangladesh | 2015 | Urine |
| *K. pneumoniae* | NDM-1 | SMU6419 | ST147 | TEM-1B + SHV-11 + SHV-12 + CTX-M-15 | Bangladesh | 2015 | Respiratory tract |
| *K. pneumoniae* | NDM-1 | SMU6421* | ST147 | TEM-209 + CTX-M-15 | Bangladesh | 2015 | Urine |
| *K. pneumoniae* | NDM-1 | SMU6423 | ST147 | TEM-1A + SHV-11 + CTX-M-15 | Bangladesh | 2015 | Urine |
| *K. pneumoniae* | NDM-1 | SMU6496 | ST147 | SHV-11 | Bangladesh | 2017 | Blood |
| *K. pneumoniae* | NDM-1 | SMU6525 | ST147 | TEM-1B + CTX-M-15 | Bangladesh | 2017 | Blood |
| *K. pneumoniae* | NDM-5 | SMU6573 | ST11 | TEM-1B + SHV-12 + CTX-M-2 | Japan | 2018 | Blood |
| *K. pneumoniae* | NDM-5 | SMU6520 | ST23 | TEM-1B + SHV-190 + CTX-M-15 + CMY-2 | Bangladesh | 2017 | Other |
| *K. pneumoniae* | NDM-5 | SMU6521 | ST23 | TEM-1B + SHV-190 + CTX-M-15 + CMY-2 | Bangladesh | 2017 | Blood |
| *K. pneumoniae* | NDM-5 | SMU6515 | ST152 | TEM-1B + CTX-M-15 | Bangladesh | 2017 | Respiratory tract |
| *K. pneumoniae* | NDM-5 | SMU6529 | ST515 | TEM-1B + SHV-145 + CTX-M-15 + CMY-16 | Bangladesh | 2017 | Respiratory tract |
| *K. pneumoniae* | NDM-5 | SMU6530 | ST515 | TEM-1B + SHV-145 + CTX-M-15 + CMY-16 | Bangladesh | 2017 | Other |
| *K. pneumoniae* | NDM-5 | SMU6424* | ST893 | TEM-1B + SHV-145 + CTX-M-15 | Bangladesh | 2015 | Other |
| *K. pneumoniae* | NDM-7 | SMU6428* | ST34 | TEM-1B + SHV-26 + CTX-M-15 + DHA-1 | Bangladesh | 2015 | Blood |
| *K. pneumoniae* | NDM-7 | SMU6570 | ST147 | SHV-12 | Japan | 2020 | Blood |
| *K. pneumoniae* | NDM-7 | SMU6467 | ST1998 | TEM-1A + CTX-M-15 | Bangladesh | 2015 | Other |
| *E. cloacae* | NDM-1 | SMU6466 | ST171 | – | Bangladesh | 2015 | Other |
| *E. cloacae* | NDM-1 | SMU6474 | ST171 | – | Bangladesh | 2015 | Other |
| *E. cloacae* | NDM-1 | SMU6592 | ST182 | TEM-1B + SHV-12 + CTX-M-15 | Japan | 2020 | Blood |
| *E. cloacae* | NDM-1 | SMU6593 | ST182 | TEM-1B | Japan | 2020 | Blood |
| *E. cloacae* | NDM-1 | SMU6589* | ST850 | TEM-1B + CTX-M-15 | Japan | 2018 | Blood |
| *E. cloacae* | NDM-1 | SMU6461 | ST1584 | TEM-1B + CTX-M-15 + DHA-1 | Bangladesh | 2015 | Urine |
| *E. cloacae* | NDM-1 | SMU6463 | ST1584 | TEM-1B + CTX-M-15 + DHA-1 | Bangladesh | 2015 | Blood |
| *E. cloacae* | NDM-1 | SMU6464 | ST1584 | CTX-M-15 + DHA-1 | Bangladesh | 2015 | Blood |
| *E. cloacae* | NDM-5 | SMU6458* | ST45 | TEM-1B + CTX-M-40 | Bangladesh | 2015 | Urine |
| *E. cloacae* | NDM-7 | SMU6590* | ST850 | – | Japan | 2018 | Blood |
| *E. cloacae* | NDM-7 | SMU6591 | ST850 | – | Japan | 2020 | Blood |

ST, sequence type; NDM, New Delhi metallo-β-lactamase.

MLST was assigned using PubMLST schemes for *E. coli* and *E. cloacae* and the Institut Pasteur BIGSdb scheme for *K. pneumoniae*.

All β-lactamase entries indicate *bla* genes; the *bla* prefix is omitted for readability.

–, no additional β-lactamase genes detected other than *bla*_NDM_.

*Isolates selected for time–kill assays and fosfomycin-resistant subpopulation analysis.
